# Effects of L-Phenylalanine on Energy Intake and Glycaemia—Impacts on Appetite Perceptions, Gastrointestinal Hormones and Gastric Emptying in Healthy Males

**DOI:** 10.3390/nu12061788

**Published:** 2020-06-16

**Authors:** Penelope C. E. Fitzgerald, Benoit Manoliu, Benjamin Herbillon, Robert E. Steinert, Michael Horowitz, Christine Feinle-Bisset

**Affiliations:** 1 Adelaide Medical School and Centre of Research Excellence in Translating Nutritional Science to Good Health, Level 5 Adelaide Health and Medical Sciences Building, Corner North Terrace and George Street, Adelaide 5005, Australia; penelope.fitzgerald@adelaide.edu.au (P.C.E.F.); benoit.manoliu@agroparistech.fr (B.M.); herbillon.b@hotmail.fr (B.H.); michael.horowitz@adelaide.edu.au (M.H.); 2 Department of Surgery, Division of Visceral and Transplantation Surgery, University Hospital Zürich, 8091 Zürich, Switzerland; steinert.robert@gmail.com

**Keywords:** food intake, appetite regulation, blood glucose control, postprandial, amino acid, cholecystokinin, peptide YY, glucagon-like peptide-1, insulin, gastric motility, humans

## Abstract

In humans, phenylalanine stimulates plasma cholecystokinin (CCK) and pyloric pressures, both of which are important in the regulation of energy intake and gastric emptying. Gastric emptying is a key determinant of postprandial blood glucose. We evaluated the effects of intragastric phenylalanine on appetite perceptions and subsequent energy intake, and the glycaemic response to, and gastric emptying of, a mixed-nutrient drink. The study consisted of two parts, each including 16 healthy, lean males (age: 23 ± 1 years). In each part, participants received on three separate occasions, in randomised, double-blind fashion, 5 g (Phe-5 g) or 10g (‘Phe-10 g) L-phenylalanine, or control, intragastrically, 30 min before a standardised buffet-meal (part A), or a standardised mixed-nutrient drink (part B). In part A, plasma CCK and peptide-YY (PYY), and appetite perceptions, were measured at baseline, after phenylalanine alone, and following the buffet-meal, from which energy intake was assessed. In part B, plasma glucose, glucagon-like peptide-1 (GLP-1), insulin and glucagon were measured at baseline, after phenylalanine alone, and for 2 h following the drink. Gastric emptying of the drink was also measured by ^13^C-acetate breath-test. Phe-10 g, but not Phe-5 g, stimulated plasma CCK (*p* = 0.01) and suppressed energy intake (*p* = 0.012); energy intake was correlated with stimulation of CCK (r = −0.4, *p* = 0.027), and tended to be associated with stimulation of PYY (r = −0.31, *p* = 0.082). Both Phe-10 g and Phe-5 g stimulated insulin and glucagon (all *p* < 0.05), but not GLP-1. Phe-10 g, but not Phe-5 g, reduced overall plasma glucose (*p* = 0.043) and peak plasma glucose (*p* = 0.017) in response to the mixed-nutrient drink. Phenylalanine had no effect on gastric emptying of the drink. In conclusion, our observations indicate that the energy intake-suppressant effect of phenylalanine is related to the stimulation of CCK and PYY, while the glucoregulatory effect may be independent of stimulation of plasma GLP-1 or slowing of gastric emptying.

## 1. Introduction

Nutrient sensing in the small intestinal lumen is critical to the release of gut and gluco-regulatory hormones, including cholecystokinin (CCK), released predominantly in the proximal small intestine, and peptide YY (PYY) and glucagon-like peptide-1 (GLP-1), in the distal small intestine [1]. These hormones mediate nutrient-induced slowing of gastric emptying, and the suppression of energy intake and postprandial blood glucose [1,2,3]. The slowing of gastric emptying, resulting in prolonged gastric distension, contributes to the perception of fullness [4], and, in the case of GLP-1, plays a critical role in the lowering of postprandial blood glucose [1]. Moreover, GLP-1 contributes to glucose lowering through its glucose-dependent effect to stimulate insulin and suppress glucagon [5].

Proteins, particularly whey protein, when administered intraduodenally or consumed 30 min before a meal, reduce energy intake and postprandial blood glucose, at least in part, by slowing gastric emptying and stimulating hormones, including CCK, PYY and GLP-1 [6,7,8]. Because relatively large amounts of protein (up to 55 g, or 226 kcal) are required for these effects, there has been an interest in characterising the effects of amino acids on upper gut functions, energy intake and postprandial blood glucose control, since amino acids are likely to mediate these effects of protein.

The aromatic amino acid, L-phenylalanine (‘phenylalanine’), is of particular interest. An earlier study found that oral ingestion of 10 g phenylalanine stimulated CCK and suppressed energy intake in healthy volunteers [9], and we reported recently that intraduodenal infusion of phenylalanine, at a load of 0.45 kcal/min for 90 min (total amount 10 g) stimulated pyloric pressures and plasma CCK [10]. Moreover, co-ingestion of 9 g phenylalanine with 25 g glucose stimulated insulin and lowered the blood glucose response to glucose, compared with the glucose-only control condition [11]. In a recent study in rats, oral gavage of phenylalanine, at doses of 3–6 mmol/kg, stimulated insulin and GLP-1, and lowered the plasma glucose response to intraperitoneal glucose [12]. However, these doses were high, corresponding to 35–70 g in a 70-kg human. Further, in murine cell cultures, 10 and 100 mM phenylalanine stimulated GLP-1, and 50 and 100 mM phenylalanine stimulated PYY [12]. Given (i) the key role of gut hormones, including CCK, GLP-1 and PYY, in the slowing of gastric emptying, (ii) the reported effects of phenylalanine to stimulate GLP-1 in preclinical studies and (iii) the role of gastric emptying as a key determinant of postprandial blood glucose, the role of gastric emptying in the reported effects of phenylalanine to lower blood glucose warrants evaluation. Moreover, the effect of phenylalanine on the release of GLP-1 and PYY in humans is, to our knowledge, unknown. Finally, since previous studies have used relatively large doses of phenylalanine (10 g), the effects of a lower dose (5 g) on these gastrointestinal (GI) functions, energy intake and postprandial blood glucose, warrant investigation. This dose represents the average phenylalanine content of a meat-based Western diet, which, when providing protein, will contain approximately 4–6 g phenylalanine [11].

The aims of this study were, therefore, to characterise the effects of intragastric phenylalanine (using doses of 5 g and 10 g) on (1) the stimulation of plasma CCK and PYY, appetite perception and energy intake from a subsequent buffet-meal and (2) gastric emptying of, and the plasma GLP-1, insulin, glucagon and glucose responses to, a mixed-nutrient drink.

## 2. Materials and Methods

### 2.1. Study Participants

Healthy, normal-weight males were included into the study, 16 (mean age: 23 ± 1 years, body mass index (BMI): 22.6 ± 0.5 kg/m^2^) in part A, and 16 (mean age: 23 ± 1 years, BMI: 21.7 ± 0.4 kg/m^2^) in part B. Five volunteers completed both study parts. Participants were recruited by advertisement from the general public and screened before their inclusion to exclude phenylketonuria, GI symptoms or a history of GI disease or surgery, vegetarians, smokers, alcohol consumption of >2 drinks (20 g ethanol) on >5 days/week, use of medications known to affect energy intake, appetite or GI function, high-performance athletes, lactose intolerance, consumption of protein supplements, unstable body weight (≥5% change over the last 3 months before participation), restrained eaters (score > 12 on the restrained eating component of the 3-factor eating questionnaire [13]), and an inability to tolerate or comprehend the study protocol. All participants provided written, informed consent prior to their inclusion. Once participants were enrolled, they were assigned to a treatment order of balanced randomisation that was generated with an online tool (www.randomization.com) by a researcher who was not involved in data analysis. The study protocol was approved by the Human Research Ethics Committee of the Central Adelaide Local Health Network and performed in accordance with the Declaration of Helsinki. The study was registered as a clinical trial with the Australian New Zealand Clinical Trials Registry (www.anzctr.org.au/Trial/Registration/TrialReview.aspx?id=371736&isReview=true; ACTRN12616001595404).

### 2.2. Study Design

**Figure 1:**
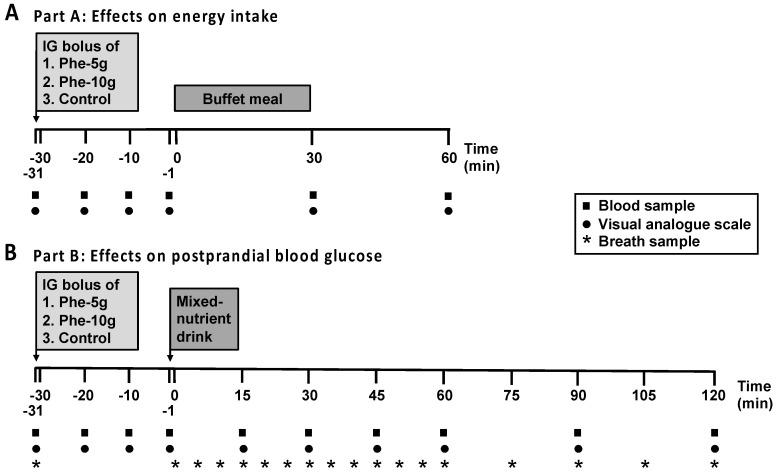
Schematic representation of the study design. (**A**): study protocol of part A. (**B**): study protocol of part B. On study days in part A, at t = −31 min, a bolus (100 mL) of phenylalanine, at doses of either 5 g (Phe-5 g) or 10 g (Phe-10 g), or control, was administered intragastrically (IG) via a nasogastric tube within 1 min, and the tube was then removed. At t = 0 min, they were presented with a standardised, buffet-style meal for the assessment of energy intake and instructed to eat until they were comfortably full, for up to 30 min. Blood samples, for the measurement of plasma cholecystokinin and peptide YY, and visual analogue scale ratings, for measurement of appetite perceptions and GI symptoms, were collected at baseline (t = −31 min) and at regular time points throughout the study, as indicated. On study days in part B, at t = −31 min, as in part A, participants received phenylalanine or control. 30 min later, at t = −1 min, participants consumed 300 mL of a mixed-nutrient drink, containing 400 kcal and 56 g carbohydrate, within 1 min. The drink was labelled with 100 mg of ^13^C-acetate for measurement of gastric emptying by ^13^CO_2_ breath test. Blood samples were collected for measurement of plasma glucose, insulin, glucagon and GLP-1 concentrations, and breath samples were collected for subsequent analysis of ^13^CO_2_, at baseline and at regular time points throughout the study, as indicated.

This study evaluated the effects of intragastric administration of L-phenylalanine, at doses of 5 g (Phe-5 g) or 10 g (Phe-10 g), or control, on (1) energy intake, plasma CCK and PYY concentrations and appetite perceptions (part A) and (2) the plasma glucose, insulin, glucagon and GLP-1 responses to, and gastric emptying of, a mixed-nutrient drink (part B) (Figure 1).

### 2.3. Study Treatments

Due to the low solubility of phenylalanine, treatments were prepared as suspensions of 5 g or 10 g phenylalanine (Healthwise, Maroochydore, Queensland, Australia) and 29 mg CaCl_2_ × 2 H_2_O in 10 mL of a suspending agent (ORA-Plus: Perrigo, Perth, Western Australia, Australia), and adjusted to a final volume of 100 mL with isotonic saline. The control consisted of 29 mg CaCl_2_ × 2 H_2_O, 10 mL of the suspending agent and isotonic saline to a final volume of 100 mL. Suspensions were prepared at room temperature on the morning of each study visit by a researcher who was not involved in data analysis, filled into syringes and administered via a nasogastric catheter directly into the stomach to avoid potential influences due to the taste of the treatments. Syringes were covered to blind both the subjects and investigator performing the study.

### 2.4. Study Protocol

In both parts A and B, subjects were studied on three occasions separated by 3–7 days, in a randomised, double-blind fashion. Participants were instructed to refrain from strenuous exercise and alcohol consumption for 24 h before each study visit and provided with a standardised meal (beef lasagne, 400 g: total energy content: 1160 kcal; McCain Food, Wendouree, Victoria, Australia) to be consumed by either 8 p.m. (part A) or 6.30 p.m. (part B) the night before each study visit, after which participants were instructed to refrain from solids and liquids, except water. Having fasted for 14 h, participants attended the Clinical Research Facility at the Adelaide Medical School, University of Adelaide, at 10 a.m. (part A) or 8.30 a.m. (part B).

On study days in part A, upon arrival, an intravenous cannula was placed in a forearm vein for regular blood sampling. Participants were then seated in an upright position and a custom-built nasogastric, soft silicon feeding tube (outer diameter: 4 mm; Dentsleeve International, Mississauga, Ontario, Canada) was inserted through an anaesthetised nostril, with the tip placed in the stomach. At t = −31 min (10.30 a.m.), participants received a 100-mL intragastric bolus of either Phe-5 g or Phe-10 g, or control, over 1 min, and the nasogastric tube was then removed. 30 min later, i.e., t = 0 min, participants were presented with a standardised, cold, buffet-style meal, and instructed to eat until they were comfortably full [14]. The participants were not made aware that the purpose of the meal was to assess energy intake. Blood samples for the measurement of gut hormone concentrations, and visual analogue scale questionnaires to assess appetite-related perceptions and GI symptoms, were collected at baseline (t = −31 min), after phenylalanine or control (t = −20, −10, −1 min), and following the buffet meal (t = 30 and 60 min) (Figure 1A) [15]. At t = 60 min, the intravenous cannula was removed, and participants were allowed to leave the laboratory.

On study days in Part B, upon arrival an intravenous cannula and a nasogastric tube were positioned, as described in part A. At t = −31 min (9 a.m.), participants received a 100-mL intragastric bolus of either Phe-5 g or Phe-10 g, or control, over 1 min, and the nasogastric tube was then removed. 30 min later, i.e., at t = −1 min, participants consumed, within 1 min, a 300-mL mixed-nutrient drink (Ensure Plus, vanilla flavour (267 mL can), Abbott Australasia Pty Ltd, Macquarie Park, New South Wales, Australia; 400 kcal, 56 g carbohydrate, 15 g protein (predominantly casein), 13 g fat, plus 33 ml water to make up the final volume) labelled with 100 mg of ^13^C-acetate for measurement of gastric emptying by breath test [16]. Blood samples, for measurement of plasma glucose and glucoregulatory hormone concentrations, and breath samples, for subsequent analysis of ^13^CO_2_ for measurement of gastric emptying, were collected at baseline (t = −31 min) and at regular time points throughout the study (Figure 1B). At t = 120 min, the intravenous cannula was removed and the participant provided with a light lunch, after which they were free to leave the laboratory.

### 2.5. Measurements

#### 2.5.1. Plasma Hormone and Glucose Analyses

Blood samples were collected into ice-chilled tubes containing ethylenediaminetetraacetic acid (for analysis of plasma CCK and PYY (part A), as well as insulin, glucagon and GLP-1 (part B) concentrations), and sodium fluoride (for plasma glucose analysis (part B)). Plasma was obtained by centrifuging samples at 1832 g-force for 15 min at 4 °C within 15 min of collection and stored at −80 °C until subsequent analysis.

Plasma CCK-8 concentrations (pmol/L) were measured after ethanol extraction using an adaptation of an established radioimmunoassay [17]. The antibody used recognises sulphated CCK-8 and does not bind to structurally unrelated peptides. Cross-reactivity with unsulphated CCK-8 was 15% and with human gastrin I 0.2%. The minimum detectable concentration was 1 pmol/L, and intra- and inter-assay coefficients of variation (CVs) were 10.9% and 13.8%, respectively.

Plasma PYY concentrations (pmol/L) were measured by radioimmunoassay using an antiserum (kindly donated by Dr. B Otto, Medizinische Klinik, Klinikum Innenstadt, University of Munich, Munich, Germany) against human PYY (1–36) (Sigma-Aldrich, St Louis, MO, USA) and raised in rabbits. This antiserum showed < 0.001% cross-reactivity with human pancreatic polypeptide or sulphated CCK-8 and 0.0025% cross-reactivity with human neuropeptide Y. The minimum detectable concentration was 1.5 pmol/L, and intra and inter-assay CVs were 14.4% and 4.3%, respectively [8].

Plasma GLP-1 concentrations (pmol/L) were measured by radioimmunoassay (GLPIT-36HK, Millipore, Billerica, MA, USA). The minimum detectable concentration was 3 pmol/L, and intra and inter-assay CVs were 7.0% and 10.2%, respectively.

Plasma insulin concentrations (mU/L) were measured using an ELISA immunoassay (10–1113, Mercodia, Uppsala, Sweden). The minimum detectable concentration was 1 mU/L, and intra and inter-assay CVs were 2.1% and 9.6%, respectively.

Plasma glucagon concentrations (pg/mL) were measured by radioimmunoassay (GL-32K, Millipore, Billerica, MA, USA). The minimum detectable concentration was 15 pg/mL, and intra and inter-assay CVs were 3.8% and 6.7%, respectively.

Plasma glucose concentrations (mmol/L) were measured by the glucose oxidase method using a glucose analyser (YSI 2900 STAT Plus; Yellow Springs Instruments, Yellow Springs, Ohio, USA).

#### 2.5.2. Measurement of Gastric Emptying

Gastric emptying was measured by breath test using ^13^C-acetate [16]. ^13^CO_2_ concentrations in end-expiratory breath samples were measured using an isotope ratio mass spectrometer (FANCi; Fischer Analysen Instrumente GmbH, Leipzig, Germany) with an online gas chromatographic purification system. The results were expressed as percentage of ^13^CO_2_ recovery/hour and profiles plotted as cumulative values over the sampling period [18].

#### 2.5.3. Appetite Perceptions, GI Symptoms and Energy Intake

Appetite-related perceptions (fullness, hunger, desire to eat and prospective food consumption) were assessed using a validated 100-mm VAS questionnaire [19]. Nausea and bloating were also assessed. Each VAS evaluated a sensation on a 100-mm horizontal line, where 0 mm represented “sensation not felt at all” and 100 mm represented “sensation felt the greatest”. Participants rated how they were feeling at a given time-point by placing a vertical stroke at the appropriate point on the line.

Energy intake at the buffet meal was calculated from the amount of food and drink (g) consumed at the meal, obtained by weighing all food items before and after being offered to the participant. The meal comprised 4 slices (120 g) of whole-meal bread, 4 slices (120 g) of white bread, 100 g sliced ham, 100 g sliced chicken, 85 g sliced cheddar cheese, 100 g lettuce, 100 g sliced tomato, 100 g sliced cucumber, 22 g mayonnaise, 20 g margarine, 1 apple (170 g), 1 banana (190 g), 175 g strawberry yogurt, 100 g chocolate custard, 120 g fruit salad, 375 mL iced coffee, 300 mL orange juice and 600 mL water, and had a total energy content of 2300 kcal (27% fat, 52% carbohydrate and 21% protein) and weight of 2924 g. Energy intake (kcal) was then calculated using commercial software (FoodWorks 8.0; Xyris Software, Highgate Hill, Queensland, Australia) [14].

### 2.6. Data and Statistical Analyses

The number of participants was determined by power calculations based on our previous studies [18,20,21]. We calculated that *n* = 16 participants would allow detection of a 205-kcal difference in energy intake (part A), and a 1.0 mmol/L reduction in plasma glucose (part B), both at α = 0.05, with a power of 80%.

To evaluate the effects of phenylalanine alone on plasma hormone and glucose concentrations and VAS ratings (part A only), data were summarised by calculating areas under the curve (AUC) from t = −31 to –1 min (AUC_−31 to −1 min_), using the trapezoidal rule. To evaluate the responses to the buffet meal, AUCs from t = −1 to 60 min (AUC_−1 to 60 min_) were calculated for plasma CCK and PYY, and VAS data. To evaluate the responses to the mixed-nutrient drink, AUCs from t = −1 to 120 min (AUC_−1 to 120 min_) were calculated for plasma glucose, insulin, glucagon and GLP-1 concentrations to characterise the overall response, while AUCs from t = −1 to 30 min (AUC_−1 to 30 min_) for plasma glucose, insulin, glucagon and GLP-1 concentrations were calculated to characterise the ‘early’ response. Peak plasma glucose was also determined. Gastric emptying data were expressed as AUCs from t = 0 to 120 min (AUC_0 to 120 min_).

Statistical analysis was performed in collaboration with a professional biostatistician, using SPSS software (version 24.0; SPSS Inc., Chicago, IL, USA). Energy intake, total amount (g) consumed and peak plasma glucose were analysed using repeated-measures one-way ANOVA with treatment (control, Phe-5 g, Phe-10 g) as a factor. All AUC data were analysed using a mixed models analysis, including baseline (t = −31 min) as a covariate and treatment as a fixed factor. An unstructured covariance matrix was used to account for repeated treatments on each subject. Model assumptions of normality and constant variance were assessed via residual plots, and where assumptions were not met, a natural logarithmic transformation was applied to the data. For all ANOVAs sphericity was evaluated by Mauchly’s test, and when violated, the Greenhouse–Geisser P value was reported. Post-hoc comparisons, adjusted for multiple comparisons using Bonferroni’s correction, were performed where ANOVAs or mixed models analyses revealed significant effects. Linear within-subject correlations were performed, in part A, between energy intake with plasma CCK and PYY concentrations, and appetite-related perceptions, at t = −1 min (i.e., directly before the buffet-meal), and, in part B, between peak plasma glucose with plasma insulin, glucagon and GLP-1 concentrations at t = −1 min (i.e., directly before the mixed-nutrient drink) and AUCs_−1 to 30 min_ (i.e., early response to the mixed nutrient drink), and with gastric emptying AUC_0 to 30 min_, as well as with the dose of phenylalanine, including baseline values as co-variates. Differences were considered statistically significant at *p* ≤ 0.05. All data are reported as means ± SEMs.

## 3. Results

All subjects completed all study visits without adverse effects. Plasma insulin data and breath test data were unavailable in one participant each, due to technical problems.

### 3.1. Part A: Effects on Energy Intake

#### 3.1.1. Appetite Perceptions and GI Symptoms

**Figure 2:**
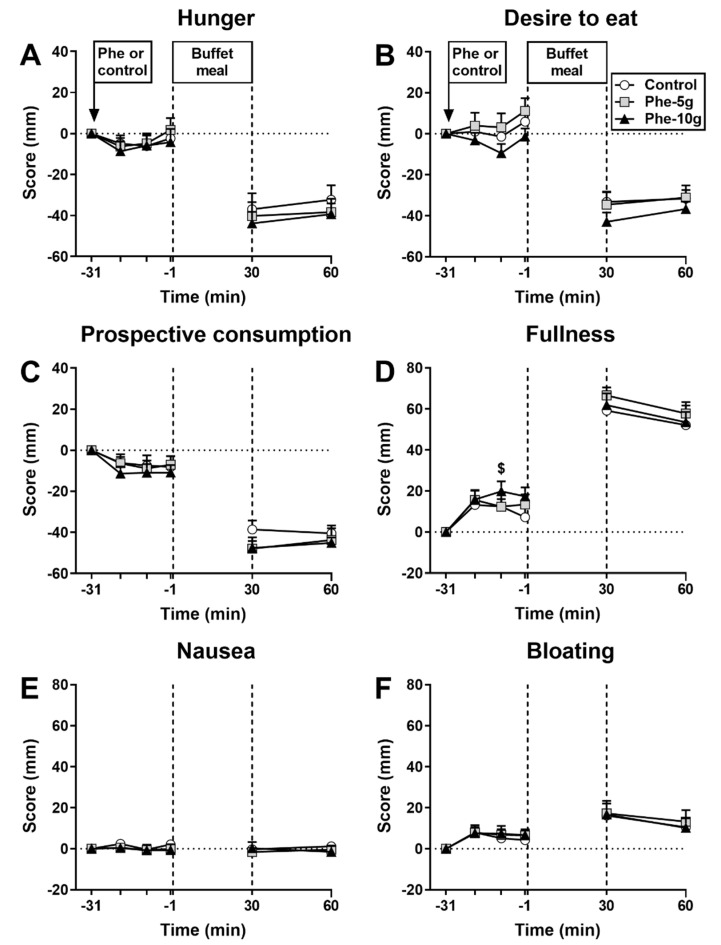
Hunger (**A**), desire to eat (**B**), prospective food consumption (**C**), fullness (**D**), nausea (**E**) and bloating (**F**) at baseline (t = −31 min), in response to an intragastric bolus (100 mL) of phenylalanine, at doses of 5 g (Phe-5 g) or 10 g (‘Phe-10 g), or control (t = −20, −10, −1 min) and after a buffet-meal (t = 30 and 60 min). Data were analysed using a mixed models analysis, including baseline as a covariate and treatment as a fixed factor. Post hoc comparisons, adjusted for multiple comparisons by Bonferroni’s correction, were conducted when the mixed models analysis revealed significant effects. (**D**) Fullness AUC_−31 to −1 min_: $ Phe-10g significantly different from control (*p* = 0.018). Data are means ± SEM and shown as changes from baseline; *n* = 16.

There was an effect of treatment on fullness AUC_−31 to −1 min_ (*p* = 0.016). Fullness was greater after Phe-10 g, but not Phe-5 g, compared with control (*p* = 0.018) (Figure 2). There were no effects on hunger, desire to eat, prospective food consumption, nausea or bloating. There were no effects on appetite perceptions or GI symptoms.

#### 3.1.2. Energy Intake

There was an effect of treatment on energy intake (kcal) (*p* = 0.005), but not the amount consumed (g). Phe-10 g, but not Phe-5 g, reduced energy intake compared with control (*p* = 0.012; Table 1).

#### 3.1.3. Plasma CCK

**Figure 3:**
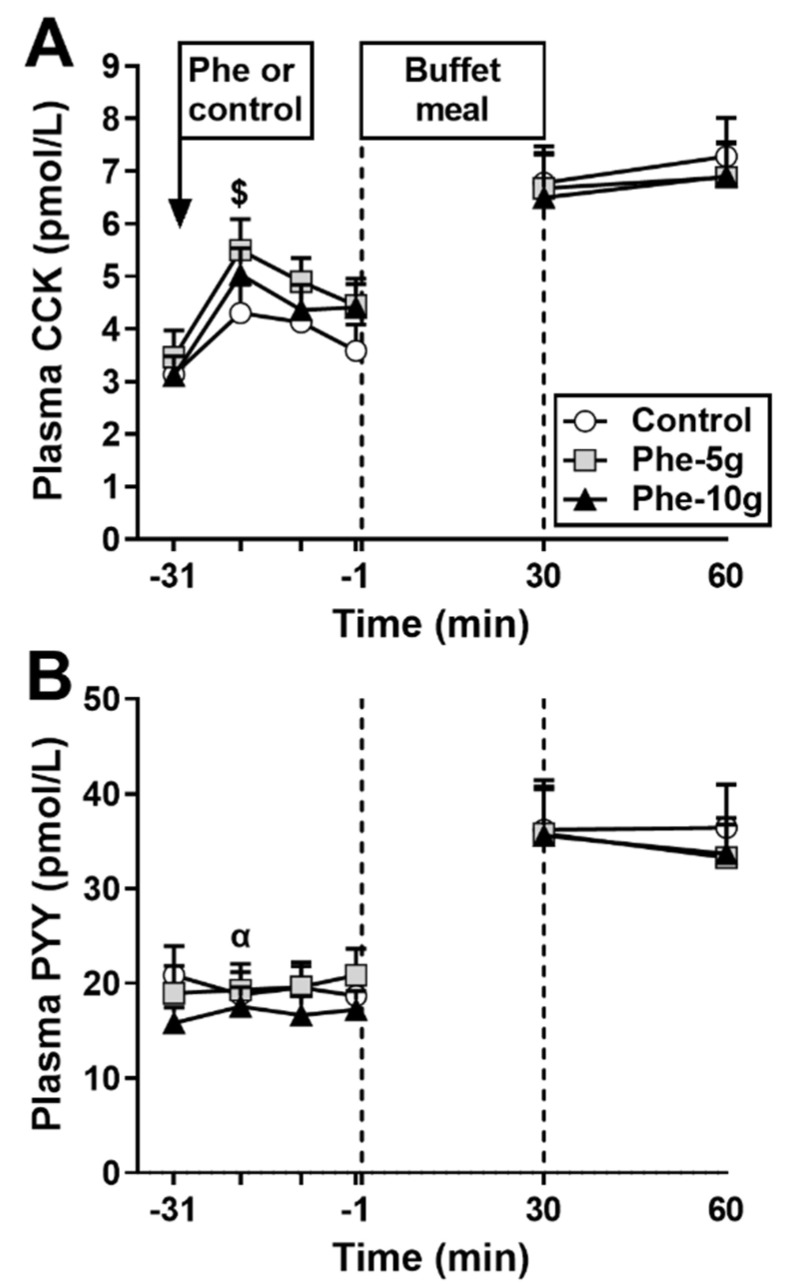
Plasma concentrations of cholecystokinin (CCK) (**A**) and peptide YY (PYY) (**B**) at baseline (t = −31 min), in response to an intragastric bolus (100 mL) of phenylalanine, at doses of either 5 g (Phe-5 g) or 10 g (Phe-10 g), or control (t = −20, −10, −1 min) and after a buffet-meal (t = 30 and 60 min). Data were analysed using a mixed models analysis including baseline as a covariate and treatment as a fixed factor. Post hoc comparisons, adjusted for multiple comparisons by Bonferroni’s correction, were conducted when the mixed model revealed significant effects. (**A**) Plasma CCK AUC_−31 to −1 min_: $ Phe-10 g significantly different from (*p* = 0.010). (**B**) Plasma PYY AUC_−31 to−1 min_: α Trend for difference for Phe-5 g compared with control (*p* = 0.074). Data are means ± SEM; *n* = 16.

There were no significant differences in fasting plasma CCK concentrations between study days (Figure 3A).

Response to phenylalanine: There was an effect of treatment on plasma CCK AUC_−31 to −1 min_ (*p* = 0.002) (Table 2), which was greater after Phe-10 g, but not Phe-5 g, compared with control (*p* = 0.010).

Response to buffet meal: Plasma CCK increased on all study days (*p* < 0.001), and remained elevated 30 min later, with no difference between treatments (*p* = 0.573).

#### 3.1.4. Plasma PYY

There were no significant differences in fasting plasma PYY concentrations between study days (Figure 3B).

Response to phenylalanine: There was a trend for an effect of treatment on plasma PYY AUC_−31 to −1 min_ (*p* = 0.057) (Table 2). Phe-5g tended to increase plasma PYY AUC_−31 to −1 min_ compared with control (*p* = 0.074).

Response to buffet meal: Plasma PYY increased on all study days (*p* < 0.01), and remained elevated 30 min later, with no difference between treatments (*p* = 0.378).

#### 3.1.5. Relationships between Dose of Phenylalanine, Energy Intake, Plasma CCK, Plasma PYY and Appetite Perceptions

There were inverse correlations between energy intake (r = –0.5, *p* = 0.001), and the amount consumed (r = −0.4, *p* = 0.001), a direct correlation between plasma CCK at t = –1 min (r = 0.5, *p* = 0.005), and a trend for a direct correlation between fullness at t = –1 min (r = 0.30, *p* = 0.060), with the dose of phenylalanine administered.

There was an inverse correlation between energy intake with plasma CCK (r = –0.4, *p* = 0.027), and a trend for an inverse correlation with plasma PYY (r = –0.31, *p* = 0.082), at t = –1 min. There were no correlations between energy intake and appetite perceptions at t = –1 min.

### 3.2. Part B: Effects on Glycaemia

#### 3.2.1. Plasma Glucose

**Figure 4:**
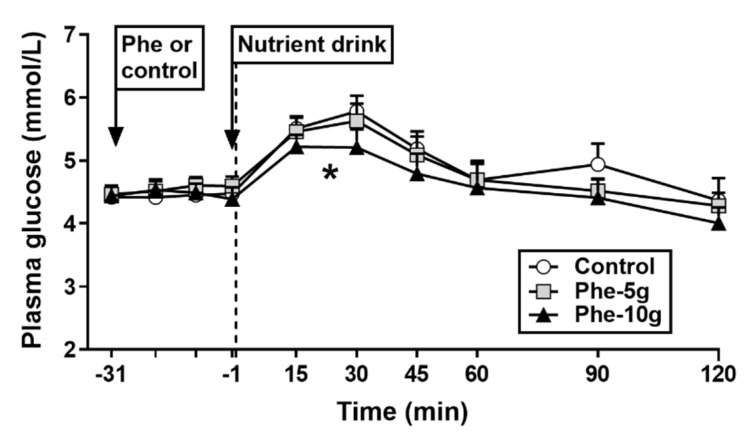
Plasma concentrations of glucose at baseline (t = 31 min), in response to an intragastric bolus (100 mL) of phenylalanine, at doses of either 5 g (‘Phe-5 g) or 10 g (‘Phe-10 g), or control (t = −20, −10, −1 min), and after a mixed-nutrient drink (t = 15–120 min). Dotted line represents the time at which consumption of the drink was complete. Data were analysed using a mixed models analysis including baseline as a covariate and treatment as a fixed factor. Post hoc comparisons, adjusted for multiple comparisons by Bonferroni’s correction, were conducted when the mixed model revealed significant effects. Glucose AUC_−1 to 120 min_: * Phe-10 g significantly different from control (*p* = 0.043). Data are means ± SEM; *n* = 16.

There were no differences in fasting plasma glucose concentrations between study days (Figure 4).

Response to phenylalanine: There was no effect of treatment on plasma glucose AUC_−31 to −1 min_ (Table 2).

Response to nutrient drink: Plasma glucose concentrations increased on all study days (*p* < 0.001). There was an effect of treatment on plasma glucose AUC_−1 to 120 min_ (*p* = 0.027). Phe-10 g, but not Phe-5 g, reduced plasma glucose AUC_−1 to 120 min_ compared with control (*p* = 0.043). There was a trend for a treatment effect on plasma glucose AUC_−1 to 30 min_ (*p* = 0.095). However, post-hoc analyses revealed no differences between treatments. There was a treatment effect on peak plasma glucose (*p* = 0.021). Phe-10 g, but not Phe-5 g, reduced peak glucose compared with control (*p* = 0.017).

#### 3.2.2. Plasma Insulin

**Figure 5:**
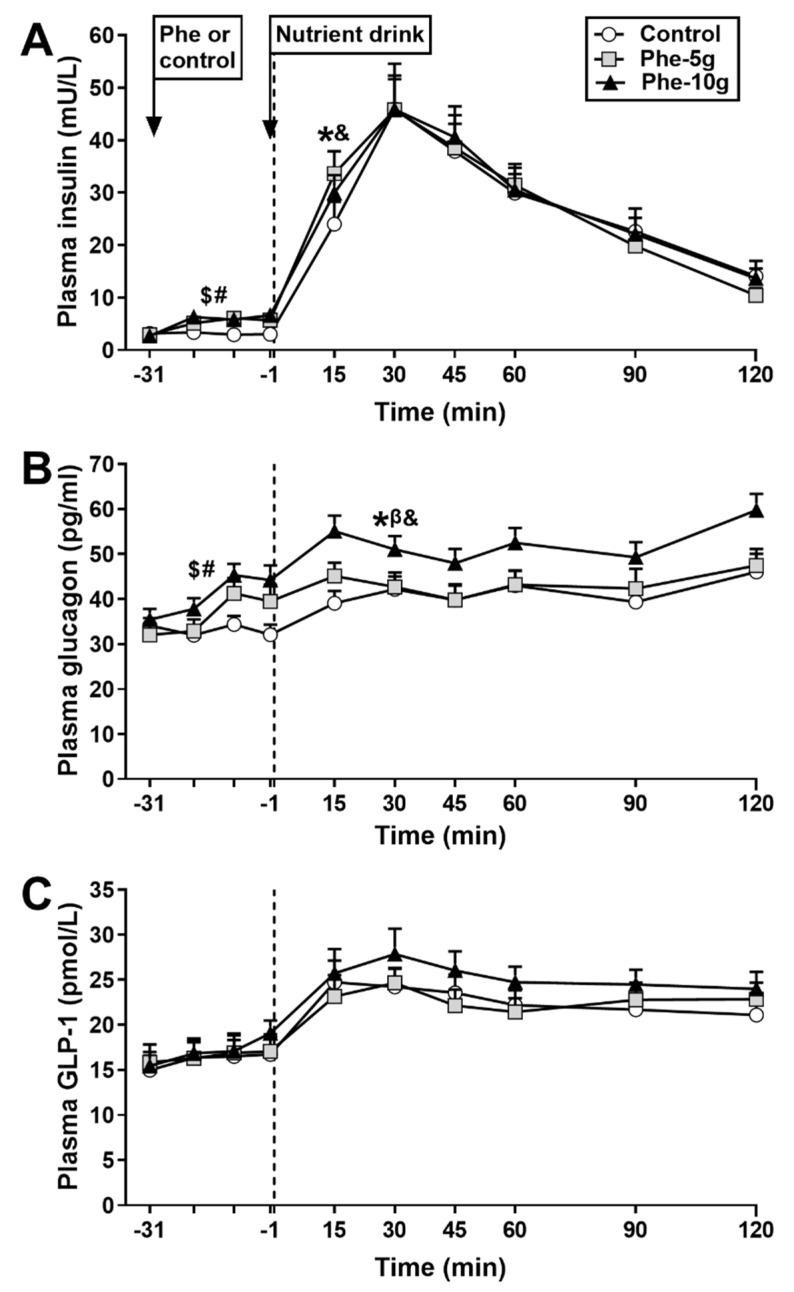
Plasma concentrations of insulin (**A**), glucagon (**B**) and glucagon-like peptide-1 (GLP-1) (**C**) at baseline (t = −31 min), in response to an intragastric bolus (100 mL) of phenylalanine, at doses of either 5 g (Phe-5 g) or 10 g (Phe-10 g), or control (t = −20, −10, 1 min), and after a mixed-nutrient drink (t = 15–120 min). Dotted line represents completion of drink consumption. Data were analysed using a mixed models analysis including baseline as a covariate and treatment as a fixed factor. Post hoc comparisons, adjusted for multiple comparisons by Bonferroni’s correction, were conducted when the mixed model revealed significant effects. (**A**) Insulin AUC_−31 to −1 min_: $ Phe-10g (*p* = 0.00) and # Phe-5 g (*p* = 0.003) significantly different from control. Insulin AUC_−1 to −30 min_: * Phe-10g (*p* = 0.006) and and Phe-5 g (*p* = 0.002) significantly different from control. (**B**) Glucagon AUC_−31 to −1 min_: $ Phe-10 g (*p* = 0.002) and # Phe-5 g (*p* = 0.003) significantly different from control. Glucagon AUC_−1 to −120 min_: * Phe-10 g (*p* = 0.002), and β trend for Phe-5 g (*p* = 0.097), compared with control. Glucagon AUC_−1 to −30 min_: * Phe-10 g (*p* = 0.001) and and Phe-5 g (*p* = 0.000) significantly different from control. Data are means ± SEM; *n* = 16, except insulin *n* = 15.

There were no differences in fasting plasma insulin concentrations between study days (Figure 5A).

Response to phenylalanine: There was an effect of treatment on plasma insulin AUC_−31 to −1 min_ (*p* = 0.000). Plasma insulin AUC_−31 to −1 min_ was greater after both Phe-10 g (*p* = 0.000) and Phe-5 g (*p* = 0.003), compared with control (Table 2).

Response to nutrient drink: Plasma insulin concentrations increased on all study days. There was no effect of treatment on plasma insulin AUC_−1 to 120 min_. There was a treatment effect for plasma insulin AUC_−1 to 30 min_ (*p* = 0.006). Both Phe-10 g (*p* = 0.006) and Phe-5 g (*p* = 0.002) increased plasma insulin AUC_−1 to 30 min_, compared with control.

#### 3.2.3. Plasma Glucagon

There were no differences in fasting plasma glucagon concentrations between study days (Figure 5B).

Response to phenylalanine: There was an effect of treatment on plasma glucagon AUC_−31 to −1 min_ (*p* = 0.000) (Table 2). Plasma glucagon AUC_−31 to −1 min_ was greater after both Phe-10g (*p* = 0.002) and Phe-5 g (*p* = 0.003), compared with control.

Response to nutrient drink: Plasma glucagon modestly increased on all study days (*p* < 0.005). There were treatment effects for plasma glucagon AUC_−1 to −120 min_ (*p* = 0.002) and AUC_−1 to −30 min_ (*p* = 0.000). Phe-10 g increased (*p* = 0.002), while Phe-5 g tended to increase (*p* = 0.097), plasma glucagon AUC_−1 to −120 min_, compared with control. Both Phe-10 g (*p* = 0.001) and Phe-5 g (*p* = 0.000) increased plasma glucagon AUC_−1 to −30 min_, compared with control.

#### 3.2.4. Plasma GLP-1

There were no differences in fasting plasma GLP-1 concentrations between study days (Figure 5C).

Response to phenylalanine: There was no effect of treatment on plasma GLP-1 AUC_−31 to −1 min_ (Table 2).

Response to nutrient drink: Plasma GLP-1 concentrations increased on all study days (*p* < 0.005). There were trends for effects of treatment on plasma GLP-1 AUC_−1 to −120 min_ (*p* = 0.087) and GLP-1 AUC_−1 to −30 min_ (*p* = 0.088). However, post-hoc analyses revealed no differences between treatments, although mean values were modestly higher after Phe-10 g.

#### 3.2.5. Gastric Emptying

**Figure 6:**
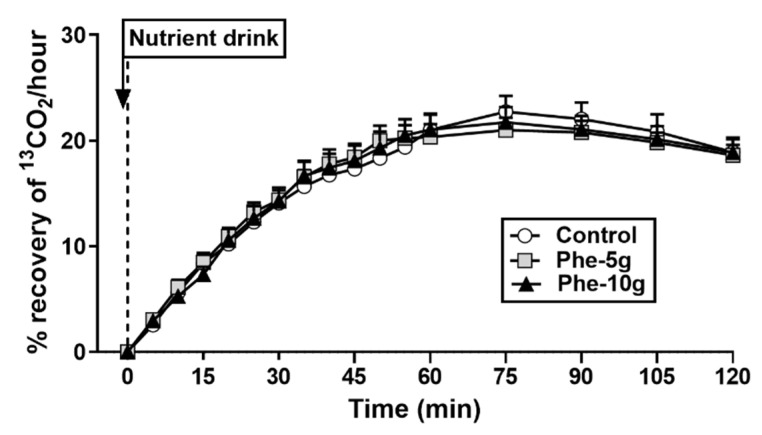
Gastric emptying of a mixed-nutrient drink (containing 100 mg [^13^C]acetate) consumed at t = −1 min, 30 min after intragastric administration of a bolus (100 mL) of phenylalanine, at doses of either 5 g (Phe-5 g) or 10 g (Phe-10 g), or control, measured by breath test and expressed as recovery of ^13^CO_2_ in breath samples. Dotted line represents the time at which consumption of the drink was complete. Data were analysed using a mixed models analysis including baseline (t = 0 min, i.e., completion of drink ingestion) as a covariate and treatment as a fixed factor. Post hoc comparisons, adjusted for multiple comparisons by Bonferroni’s correction, were conducted when the mixed model revealed significant effects. Data are means ± SEM; *n* = 15.

There was no effect of treatment on gastric emptying of the mixed-nutrient drink (Figure 6).

#### 3.2.6. Relationships between Dose of Phenylalanine, Peak Plasma Glucose, Insulin, Glucagon and GLP-1

There was an inverse correlation between peak plasma glucose (r = −0.46, *p* = 0.007), and direct correlations between insulin at t = −1 min (r = 0.70, *p* = 0.000) and glucagon at t = −1 min (r = 0.60, *p* = 0.000), with the dose of phenylalanine administered.

There was a trend for an inverse correlation between peak plasma glucose with insulin (r = −0.31, *p* = 0.1) at t = −1 min. There were no correlations between peak plasma glucose with the AUC_0 to 30 min_ for gastric emptying or AUC_−1 to 30 min_ for insulin or GLP-1.

## 4. Discussion

We have demonstrated that phenylalanine has both energy intake-suppressant and blood glucose-lowering effects in healthy people. Key findings were that (i) 10 g (Phe-10 g), but not 5 g (Phe-5 g), phenylalanine reduced energy intake at a buffet meal, (ii) Phe-10 g stimulated plasma CCK, and (iii) energy intake was related inversely to plasma CCK, and there was a trend for an inverse relationship with PYY, immediately prior to the meal, (iv) both Phe-5 g and Phe-10 g stimulated insulin and glucagon, (v) Phe-10 g, but not Phe-5 g, reduced plasma glucose in response to a mixed-nutrient drink and (vi) phenylalanine had no effect on gastric emptying. Accordingly, the reduction in energy intake is, at least in part, mediated by CCK and PYY, and blood glucose lowering is primarily mediated by insulin, while gastric emptying or GLP-1 do not appear to play a role.

Phe-10g reduced energy intake from a buffet meal consumed 30 min later by a substantial 184 kcal (17%), in the absence of nausea. While the effect of Phe-5 g was not significant, the mean caloric reduction was 100 kcal, and energy intake was related inversely to the dose of phenylalanine given. Our findings confirm, and extend, those from an earlier study [9], which reported that oral ingestion of 10 g phenylalanine, 20 min before a meal (mince meat and rice, and cake) reduced energy intake from that meal by 498 kcal (31%). The difference in the magnitude of the effect on energy intake may be due to the timing of meal ingestion; in the previous study meal consumption 20 min after phenylalanine ingestion may have been closer to peak CCK, possibly explaining the more modest energy intake suppression in response to Phe-10 g, and the lack of a significant effect of Phe-5 g, in our study.

Other gut hormones, particularly PYY and GLP-1, also play a role in the regulation of acute energy intake [22,23]. These are released predominantly from the distal small intestine, thus, their release is more delayed compared with that of CCK [24]. Indeed, we found no effect of phenylalanine alone on PYY, although there was a trend for an inverse correlation between energy intake and plasma PYY immediately before the meal, suggesting some contribution to the effects of phenylalanine on energy intake. While phenylalanine alone did not stimulate GLP-1 (as shown in part B of the study), the marked stimulation of, particularly, glucagon and, to a more moderate extent, insulin within 30 min of phenylalanine administration may suggest that these hormones also contributed to the lowering of energy intake [25,26]. However, the contribution of each hormone individually was most likely modest. Our data also do not confirm findings from preclinical studies of strong stimulatory effects on PYY and GLP-1 [12], probably because much higher doses were used in these, suggesting that, in humans, phenylalanine is not a strong PYY or GLP-1 secretagogue, also considering the relatively high doses we used.

A second aim of our study was to investigate the effects of phenylalanine on postprandial blood glucose and potential underlying GI mechanisms, particularly the role of gastric emptying, given earlier reports that phenylalanine stimulates CCK [9,10], a key regulator of the slowing of gastric emptying [27], and the reports in preclinical studies of potent effects on the incretin hormone, GLP-1 [12]. Phe-10 g lowered plasma glucose in response to a mixed-nutrient drink consumed 30 min later, by 0.6 mmol/L, compared with control. In contrast, Phe-5 g did not have an effect, although peak plasma glucose was inversely related to the dose of phenylalanine. Thus, a relatively high load of phenylalanine is required for a modest effect on blood glucose lowering. In an earlier study, phenylalanine, co-administered at a dose of 1 mmol/kg lean body mass (9 g in a 70 kg person) with 25 g glucose, did not reduce peak plasma glucose compared with glucose alone, but was associated with an earlier return of plasma glucose to baseline levels, suggesting that consumption prior to a meal may be necessary for glucose lowering. That the magnitude of glucose lowering in our study was modest may, in part, reflect the fact that the nutrient drink also contained fructose-based carbohydrates, thus, a drink with a higher content of glucose-based carbohydrates may have led to greater changes in both blood glucose and glucose lowering. The glucose-lowering effect of phenylalanine is likely to be more pronounced in people with type 2 diabetes with much more elevated postprandial glucose concentrations, and lower doses may be sufficient, which warrants investigation.

Both Phe-5 g and Phe-10 g stimulated insulin prior to the nutrient drink, i.e., in the absence of glucose. It is likely that circulating phenylalanine may have acted directly on pancreatic beta-cells to stimulate insulin. In support, phenylalanine has been reported to stimulate insulin from isolated beta-cells and insulin-secreting pancreatic beta-cell lines via the sulphonylurea receptor [28]. That insulin contributed to glucose lowering is supported by the observation that peak glucose tended to be inversely related to insulin levels immediately before the drink. Moreover, the early insulin response to the drink was greater after Phe-5 g and Phe-10 g compared with control. Both Phe-5 g and Phe-10 g also potently stimulated plasma glucagon, both before and following the mixed-nutrient drink, and this may have counteracted a more potent plasma glucose-lowering effect.

In contrast to preclinical studies [12], plasma GLP-1 was not stimulated measurably by phenylalanine alone in our study, except for a possible indication of a rise immediately before the nutrient drink, but slightly increased following the mixed-nutrient drink, with mean levels being modestly higher following Phe-10 g. Since the insulinotropic effect of GLP-1 has been reported to require glucose levels of 7–8 mmol/L [29], GLP-1-dependent stimulation of insulin was most likely not the mechanism by which glucose was lowered in our study, although we cannot exclude an effect of GLP-1 near the location of its secretion.

Gastric emptying is a major determinant of the blood glucose response to a meal [30,31]. In our study, phenylalanine did not slow gastric emptying. This was unexpected given previous reports that phenylalanine stimulates CCK, which is known to slow gastric emptying [27]. Although we did not measure CCK in this part of the study, the rise in plasma CCK in part A, although significant, was modest (2 pmol/L) and concentrations were returning towards baseline immediately before consumption of the nutrient drink, thus, potentially not reaching levels required for an effect on gastric emptying. Nevertheless, it is clear that slowing of gastric emptying did not underlie the glucose-lowering effect of phenylalanine.

A major strength of our study was the comprehensive investigation of both intake and gluco-regulatory effects of phenylalanine, and associated GI mechanisms, in humans. Some limitations should also be recognised. We only included healthy young males because they have been reported to be more sensitive to dietary manipulations [32] and to avoid any confounding influences of the menstrual cycle on GI functions and energy intake [15], therefore, our observations should not be extrapolated to females or other groups, including older people, people with obesity or type 2 diabetes. We did not analyse the effects of phenylalanine on plasma ghrelin concentrations, given that the effects on the gut hormones measured were modest. We also did not measure plasma phenylalanine, which has been related to a decreased desire to eat [33], and is a marker of levels of the neurotransmitter, dopamine, which plays a role in food reward [34,35]. Plasma phenylalanine concentrations would also provide an indication of phenylalanine absorption, however, a previous study reported substantially elevated circulating phenylalanine concentrations for about 150 min after consumption [11], suggesting a prolonged presence of phenylalanine in the GI lumen. Finally, while we measured a number of GI functions that play important roles in the regulation of appetite, energy intake and blood glucose control, it is important to recognise that the regulation of appetite and energy intake, in particular, is complex, and many factors, including the microbiome, circulating metabolites and activation of central pathways, such as the leptin-melanocortin pathway, amongst others, also play a role.

## 5. Conclusions

The findings from this study add to a growing body of evidence that a number of amino acids have effects to reduce energy intake and/or postprandial blood glucose in humans, at least in part, by stimulating the release of gut and/or pancreatic hormones, and some amino acids also by slowing gastric emptying [9,10,11,18,20,21,36,37,38]. The current study shows that phenylalanine, when given in isolation in a concentrated form, has the capacity to both reduce energy intake and lower postprandial blood glucose in healthy men in a dose-related fashion. The suppression of energy intake most likely involved a number of gut, and possibly pancreatic, hormones, while the blood glucose-lowering effect was due to insulin, while slowing of gastric emptying, surprisingly, played no role, and the involvement of GLP-1 remains uncertain. While the effects were modest, particularly considering the relatively large loads required for these effects, the data provide a rationale for establishing the appetite-suppressant and blood glucose-lowering effects of phenylalanine in people with obesity and/or type 2 diabetes, as these are the target populations for novel strategies to reduce energy intake and lower postprandial blood glucose. Such studies would need to evaluate whether the effects observed in healthy people in the current study are maintained in people with obesity and/or type 2 diabetes. Moreover, since people with type 2 diabetes have markedly elevated postprandial blood glucose levels, the magnitude of the effect of phenylalanine on blood glucose may be enhanced, thus, smaller loads may be sufficient; this warrants investigation.

## Figures and Tables

**Table 1:** Energy intake (kcal) and amount consumed (g) at an ad libitum buffet meal offered 30 min after intragastric infusion of phenylalanine, at doses of 5 g (Phe-5 g) or 10 g (Phe-10 g), or control^1.^

Variables	Control	Phe-5 g	Phe-10 g	*p*
Energy intake (kcal)	1084 ± 76	987 ± 82	900 ± 78 *	0.005
Amount consumed (g)	1096 ± 87	1065 ± 93	988 ± 78	NS

^1^ Data are means ± SEMs. *n* = 16. NS, not significant. Data were analysed using repeated-measures one-way ANOVA with treatment as a factor. Post-hoc comparisons, adjusted for multiple comparisons by Bonferroni’s correction, were conducted where ANOVAs revealed significant effects. * Significantly different from control (*p* < 0.05).

**Table 2:** Plasma CCK and PYY concentrations (part A), and plasma glucose, insulin, glucagon and GLP-1 responses to, and gastric emptying of, a mixed-nutrient drink (part B), after intragastric infusion of phenylalanine, at doses of 5 g (Phe-5 g), or 10 g (Phe-10 g), or control^1.^

Variables	Control	Phe-5 g	Phe-10 g	*p* (Mixed Models Analysis)
PART A: Effects on Energy Intake Plasma CCK				
AUC_−31 to −1_, pmol/L × min	121 ± 5	137 ± 6	135 ± 6 *	0.002
Plasma PYY				
AUC_−31 to −1_, pmol/L × min	521 ± 24	577 ± 16 γ	578 ± 30	0.057
PART B: Effects on glycaemia Plasma glucose				
AUC_−31 to −1_, mmol/L × min	134 ± 2	136 ± 2	134 ± 3	NS
AUC_−1 to 120_, mmol/L × min	601 ± 25	581 ± 20	556 ± 28 *	0.027
AUC_−1 to 30_, mmol/L × min	160 ± 4	158 ± 4	150 ± 4	0.095
Peak glucose, mmol/L	6.1 ± 0.2	5.9 ± 0.2	5.5 ± 0.3 *	0.021
Plasma insulin				
AUC_−31 to −1_, mU/L × min	89 ± 5	155 ± 16 *	173 ± 18 *	0.000
AUC_−1 to 120_, mU/L × min	3183 ± 375	3270 ± 334	3372 ± 293	NS
AUC_−1 to 30_, mU/L × min ^2^	6.4 ± 0.2	6.7 ± 0.1 *	6.6 ± 0.1 *	0.006
Plasma glucagon				
AUC_−31 to −1_, pg/mL × min	989 ± 38	1139 ± 51 *	1196 ± 39 *	0.000
AUC _−1 to 120_, pg/mL × min	4879 ± 342	5282 ± 329 §	6100 ± 304 *	0.002
AUC_−1 to 30_, pg/ mL × min	1138 ± 61	1334 ± 67 *	1507 ± 76 *	0.000
Plasma GLP-1				
AUC_−31 to −1_, pmol/L × min	499 ± 30	484 ± 16	511 ± 23	NS
AUC_−1 to 120_, pmol/L × min	2703 ± 157	2662 ± 151	2986 ± 163	0.087
AUC_−1 to 30_, pmol/L × min	688 ± 44	651 ± 31	737 ± 48	0.088
Gastric emptying				
AUC_0 to 120_, % recovery of ^13^CO_2_	2032 ± 138	2021 ± 103	2021 ± 126	NS

^1^ Data are means ± SEMs; *n* = 16, except plasma insulin and gastric emptying *n* = 15. AUC, area under the curve; CCK, cholecystokinin; GLP-1, glucagon-like peptide-1; NS, not significant; PYY, peptide tyrosine tyrosine. ^2^ Data are reported as the natural log of AUC data. Data were analysed using a mixed models analysis, including baseline (t = −31 min) as a covariate and treatment as a fixed factor. Post-hoc comparisons, adjusted for multiple comparisons using Bonferroni’s correction, were performed where mixed models analysis revealed significant effects. * Significantly different from control (*p* < 0.05). γ Trend for difference from control (*p* = 0.074). § Trend for difference from control (*p* = 0.097).
